# AI-Assisted Rational Design and Activity Prediction of Biological Elements for Optimizing Transcription-Factor-Based Biosensors

**DOI:** 10.3390/molecules29153512

**Published:** 2024-07-26

**Authors:** Nana Ding, Zenan Yuan, Zheng Ma, Yefei Wu, Lianghong Yin

**Affiliations:** 1 State Key Laboratory of Subtropical Silviculture, Zhejiang A&F University, Hangzhou 311300, China; yuanzenan20001003@163.com; 2 Zhejiang Provincial Key Laboratory of Resources Protection and Innovation of Traditional Chinese Medicine, Zhejiang A&F University, Hangzhou 311300, China; 3 Zhejiang Provincial Key Laboratory of Biometrology and Inspection & Quarantine, College of Life Sciences, China Jiliang University, Hangzhou 310018, China; mazheng520@163.com; 4 Zhejiang Qianjiang Biochemical Co., Ltd., Haining 314400, China; yefei0906@163.com

**Keywords:** synthetic biology, biological elements, transcription-factor-based biosensor, artificial intelligence, machine learning, deep learning

## Abstract

The rational design, activity prediction, and adaptive application of biological elements (bio-elements) are crucial research fields in synthetic biology. Currently, a major challenge in the field is efficiently designing desired bio-elements and accurately predicting their activity using vast datasets. The advancement of artificial intelligence (AI) technology has enabled machine learning and deep learning algorithms to excel in uncovering patterns in bio-element data and predicting their performance. This review explores the application of AI algorithms in the rational design of bio-elements, activity prediction, and the regulation of transcription-factor-based biosensor response performance using AI-designed elements. We discuss the advantages, adaptability, and biological challenges addressed by the AI algorithms in various applications, highlighting their powerful potential in analyzing biological data. Furthermore, we propose innovative solutions to the challenges faced by AI algorithms in the field and suggest future research directions. By consolidating current research and demonstrating the practical applications and future potential of AI in synthetic biology, this review provides valuable insights for advancing both academic research and practical applications in biotechnology.

## 1. Introduction

Biological elements (bio-elements) are the fundamental building blocks of synthetic biological systems, consisting of molecular sequences with specific functions. These bio-elements include nucleic acids, such as promoters, enhancers, and ribosome binding sites (RBS), as well as proteins, such as transcription factors (TF) and enzymes. The rapid development of synthetic biology has made the rational design of bio-elements and the precise prediction of their activity key areas of research [1,2,3,4,5]. However, the sequences and functions of bio-elements are complex, and their intrinsic relationships remain unclear. Traditional methods for designing bio-elements and predicting their activity often suffer from being time-consuming, costly, unreliable, lacking clear optimization directions, and having insufficient relevant biological theories. Fortunately, the rapid development of machine learning algorithms in artificial intelligence (AI), such as support vector machines, logistic regression, and decision trees, combined with big data, has shown excellent performance in various fields [6,7,8,9,10]. Machine learning possesses strong capabilities to understand biological data and make autonomous decisions, enabling the extraction of hidden features within biological data that are difficult to obtain through experimental methods [11,12]. Deep learning [13], a new branch of machine learning, can efficiently learn latent patterns in data [14,15]. For example, convolutional neural networks (CNNs, a type of feed-forward neural network that incorporates convolutional calculations and has a deep architecture) and recurrent neural networks (RNNs, a type of neural network designed to take sequence data as input and recursively process it in the order of the sequence) can achieve precise predictions of bio-element activity by learning sequence features [16,17,18,19,20]. Generative adversarial networks (GANs, a type of generative deep learning model that consists of two competing components, a generator and a discriminator, which are trained simultaneously to generate realistic synthetic data) can achieve de novo design of bio-elements through adversarial training between generators and discriminators [21,22,23,24]. Therefore, AI-guided de novo design and precise activity prediction of bio-elements present new opportunities for bio-element research.

The rational design and activity prediction of bio-elements are crucial steps in synthetic biology research. Activity prediction results provide reference standards and optimization directions for designing bio-elements with ideal performance. Simultaneously, bio-elements obtained through rational design must be evaluated for reliability by predicting and verifying their performance. Therefore, the rational design and activity prediction of bio-elements are often complementary in the development of synthetic biological systems. However, the design of bio-elements based on the “Design–Build–Test–Learn” (DBTL) cycle often relies on extensive experimental trial and error and still lacks efficient characterization methods. In addition, traditional methods for predicting the activity of bio-elements, which explore the “genotype–phenotype” relationship, are often complex, low in accuracy, and highly dependent on relevant biological mechanisms and theories such as thermodynamics and molecular dynamics [3,25,26]. To address these challenges, AI algorithms have garnered widespread attention from researchers [27,28,29,30]. For example, Wang et al. achieved the de novo design of *E. coli* promoters using a GAN model [31]. Jores et al. employed a CNN model to accurately predict promoter activity and rationally designed plant promoters with the desired activity [32]. In these studies, AI algorithms effectively simplified the DBTL exploration process, reduced experimental costs, and provided new directions for the design and optimization of nucleic acid elements. Additionally, deep-learning-based models such as AlphaFold have been used to predict protein structures and characteristics, offering new references for the rational design of protein elements [33,34].

**Figure 1:**
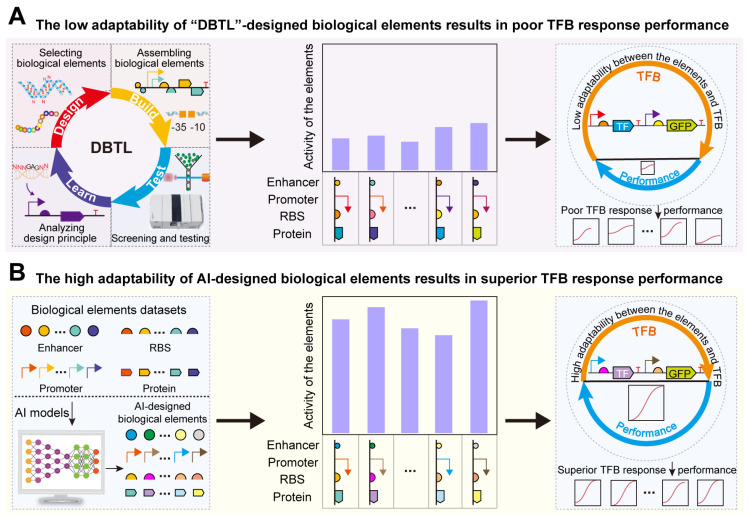
Design of biological elements to regulate the TFB response performance: (**A**) The low adaptability of “DBTL”-designed biological elements results in poor TFB response performances. (**B**) The high adaptability of AI-designed biological elements results in superior TFB response performances.

One significant application of de novo-designed bio-elements is their efficient and precise adaptation for optimal response performance in transcription factor-based biosensors (TFBs) [35,36]. TFBs can convert target metabolite concentration signals into fluorescence or expression-level signals of metabolic pathways [37]. They have been widely used in target metabolite concentration detection [38], high-throughput screening [39], direction evolution [40], and dynamic regulation [41,42]. To enhance the robustness and reliability of TFB applications, it is necessary to design TFBs with excellent response performance. The evaluation metrics for TFB response performance include dynamic range, detection range, specificity, and sensitivity. However, bio-elements designed using traditional “DBTL” methods often fail to achieve optimal TFBs, commonly exhibiting issues such as inappropriate dynamic range, low sensitivity, and low specificity [35,43] (Figure 1A). Thus, designing bio-elements that are highly compatible with TFBs to optimize their response performance remains a significant challenge. Previous studies have utilized the rational design of bio-elements, such as promoters, RBS, and TFs, to regulate TFB response performance. For instance, d’Oelsnitz et al. constructed a TFB with two CamR binding sites, doubling its dynamic range [44]. Gong et al. introduced mutations to modify the structure of the transcription factor TrpR, increasing the specificity of TFB for tryptophan by over threefold compared with 5-hydroxytryptophan [45]. However, designing bio-elements using traditional experimental methods is not only time-consuming and labor-intensive but also limits further optimization of TFB response performance. AI algorithms, with their ability to uncover underlying rules, are promising technologies for regulating TFB response performance (Figure 1B). For example, Ding et al. trained a CNN on large datasets associating RBS and TFB dynamic range, constructing a classification model CLM-RDR to achieve intelligent regulation of TFB dynamic range [46]. This demonstrates that AI algorithms can bypass some complex biological mechanisms to quickly and accurately optimize TFB response performance. Therefore, AI-designed bio-elements can be adapted to achieve TFBs with excellent response performance. 

In this review, we highlight the recent applications and challenges of machine learning and deep learning models in the rational design and activity prediction of bio-elements. We also explore how AI-designed bio-elements can regulate the TFB response performance. By outlining the advantages, adaptability, and biological challenges addressed by AI algorithms, we aim to assist researchers in leveraging AI for bio-element-related research. Furthermore, we propose innovative solutions to overcome these challenges and suggest future research directions. Detailed comparisons of traditional and AI-based methods, their accuracy, and the challenges they address are summarized in Table 1, providing a comprehensive understanding of the current state and future potential of AI in synthetic biology. 

## 2. AI-Based Rational Design and Activity Prediction of Bio-Elements

**Figure 2:**
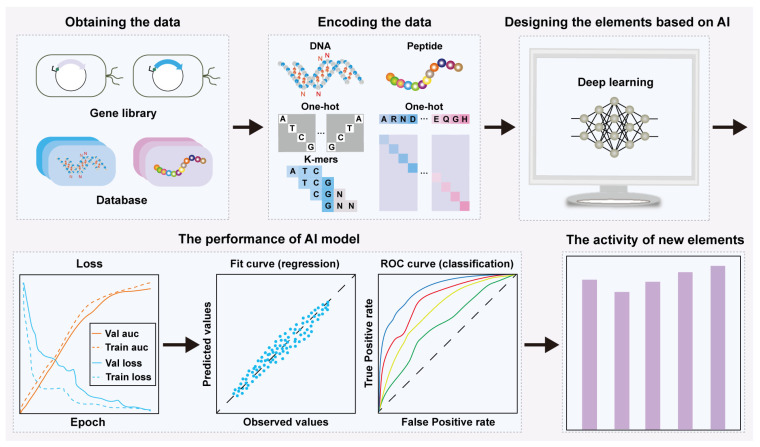
AI-designed biological elements and their activity prediction. Loss: an indicator for evaluating the training process and testing process of the model; Val and Train auc: an index used to evaluate the model accuracy of the validation process and training process, respectively; Val and Train loss: an index used to evaluate the loss of model validation process and training process, respectively; ROC and Fit curve: indicators for evaluating the performance of the classification and regression model, respectively.

The rational design and activity prediction of bio-elements are crucial research areas in synthetic biology. AI algorithms have been widely applied to the rational design and precise activity prediction of genetic regulatory elements such as promoters and RBSs (Figure 2), as well as the intelligent design of protein sequences and structures. Combining AI models with traditional biological experiments will enhance the depth of research into bio-elements.

### 2.1. AI-Assisted Rational Design and Activity Prediction of Promoters

**Figure 3:**
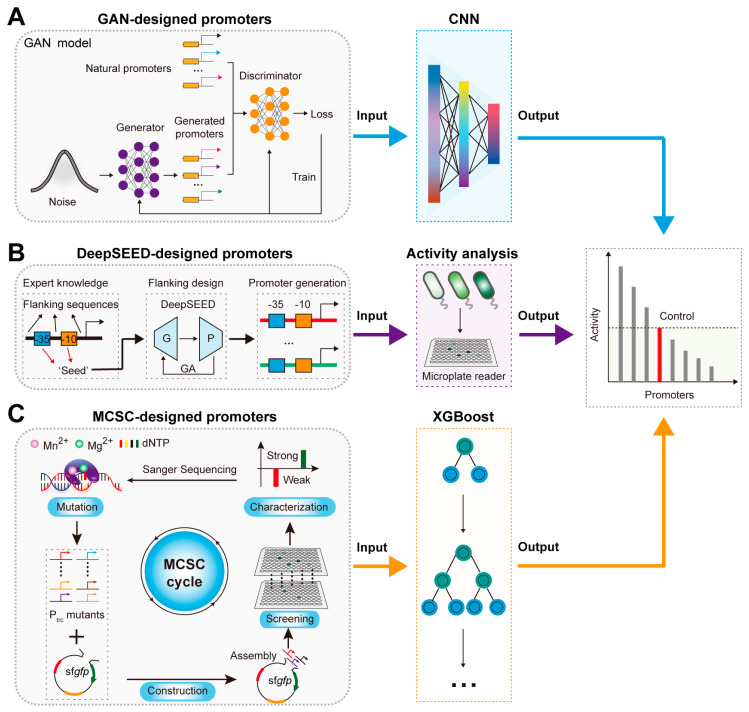
AI–derived promoter design and activity prediction: (**A**) The activity prediction of GAN–designed promoters based on the CNN model. (**B**) The activity prediction of DeepSEED–designed promoters based on activity analysis. G, P, and GA represent the Generator, Predictor, and Genetic algorithm. (**C**) The activity prediction of MCSC–designed promoters based on the XGBoost model.

Promoters are short DNA sequences located near gene transcription sites specifically recognized and bound by RNA polymerase to initiate transcription, playing a crucial role in regulating gene expression levels [61]. The rational design and activity prediction of promoters are playing a crucial role in synthetic biology and metabolic engineering. However, promoter design faces challenges such as a small library and a vast potential sequence space [31,62]. To address these challenges, AI models have been employed [63]. For example, Wang et al. achieved the de novo design of *E. coli* promoter sequences using a GAN model (Figure 3A) [31]. They first employed adversarial training between the generator and discriminator in a GAN model to learn and extract the latent features of natural promoters, generating a large number of novel artificial promoter sequences [31]. Finally, they predicted the activity of these artificial promoters based on a CNN model, achieving a prediction accuracy of 0.7, demonstrating the excellent performance of AI-designed artificial promoters [31]. Moreover, Zhang et al. developed the DeepSEED framework based on GAN (generator) and Long Short-Term Memory (LSTM, a predictor, is a specialized type of recurrent neural network designed to effectively capture long-term dependencies in sequential data) models, improving the prediction accuracy of promoter activity to 0.78 by considering the influence of flanking sequences during the design process (Figure 3B) [47]. 

Predicting promoter activity also faces challenges such as high costs and low accuracy. To address these issues, Zhao et al. proposed a promoter strength prediction method based on the eXtreme Gradient Boosting model (XGBoost, a powerful machine learning algorithm based on decision trees, optimized for efficiency, accuracy, and handling large datasets through gradient boosting techniques) (Figure 3C) [48]. By constructing a gradient transcription intensity Trc promoter (Ptrc) mutant library dataset based on the mutation–construction–screening–characterization (MCSC) cycle, the XGBoost model was trained, achieving a prediction accuracy of 0.88 [48]. Similarly, Qiao et al. developed the iPro-GAN model, which used spatial data analysis to extract the sequence features based on the Moran model and employed the deep convolutional GAN model to achieve high-precision predictions of promoter transcription intensity, ultimately reaching a prediction accuracy of 0.92 [49]. These examples demonstrate AI algorithms’ potential in the rational design and precise activity prediction of promoters.

### 2.2. AI-Assisted Rational Design and Activity Prediction of Enhancers

**Figure 4:**
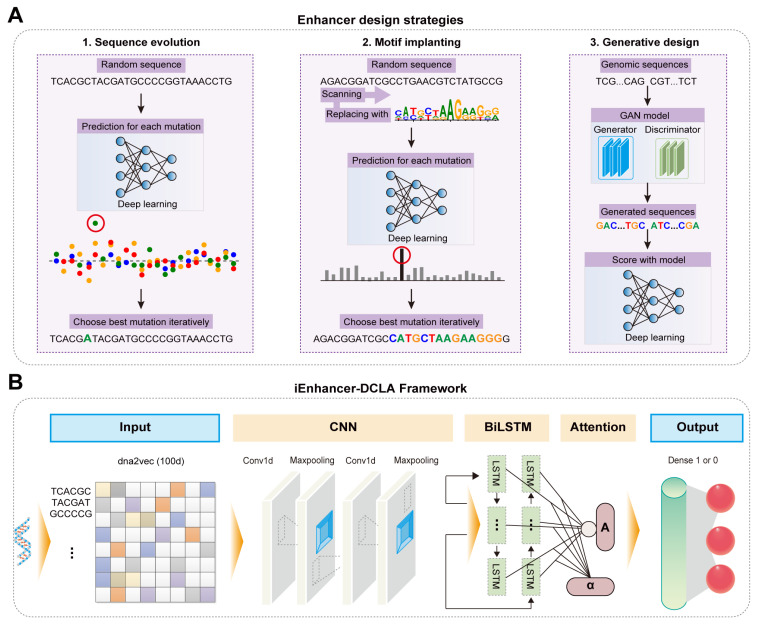
AI-derived enhancer design and activity prediction: (**A**) The overview of enhancer design strategies based on deep learning. (**B**) The model structure of enhancer activity prediction based on iEnhancer-DCLA. It includes feature representation based on the dna2vec method, two convolutional and maxpooling layers, a bidirectional LSTM network layer, an attention layer, and two fully connected layers. A represents the feature vector after passing through the attention mechanism layer; α represents the importance of the output of the bidirectional LSTM layer.

Enhancers, typically containing multiple motifs such as transcription factor binding sites (TFBS), are short regions within eukaryotic sequences that can bind to proteins and enhance gene transcription, playing a crucial role in regulating gene expression [64,65]. However, the de novo design and activity prediction of enhancers is challenging due to the unclear relationship between motif syntax and enhancer activity, the inadequate compatibility between motifs, and the limited applicability [66]. To solve these challenges, Almeida et al. developed DeepSTARR, a CNN-based deep learning framework, achieving efficient prediction of enhancer activity in Drosophila S2 cells with a Pearson Correlation Coefficient (PCC, a statistical measure that evaluates the strength and direction of the linear relationship between two variables, often used to assess the performance of a model by comparing predicted values to actual outcomes) of 0.74 [50]. Experimental validation based on the prediction results revealed some motif syntax rules. Using these rules, they designed novel enhancer sequences with gradient activities ranging from 0.8 to 630 [50]. Taskiran et al. further realized the de novo design of synthetic enhancers based on cell types using various AI algorithms (Figure 4A) [67]. First, they used AI algorithms for directed sequence optimization and the insertion of TFBS into enhancers, analyzing the impact of changes in repressive sites and TFBS on enhancer activity [67]. Then, they compared Drosophila enhancers designed using these strategies with human enhancers generated by a GAN model, demonstrating the applicability of these design strategies to different biological systems [67]. In addition, Liao et al. developed iEnhancer-DCLA, a novel deep learning framework, to predict enhancer activity (Figure 4B) [51]. First, they encoded sequences using data encoding methods such as word embedding, one-hot encoding, and k-mers to determine the most suitable approach for enhancer sequences [51]. Then, they combined algorithms like CNN, LSTM, and attention mechanisms to thoroughly extract sequence features [51]. The model achieved an accuracy of 0.83 in predicting enhancer activity. Thus, AI algorithms have shown significant potential in the rational design and precise activity prediction of enhancers [68,69].

### 2.3. AI-Assisted Rational Design and Activity Prediction of RBS

RBS is an untranslated region upstream of the mRNA start codon, recognized and bound by the ribosome to initiate translation. RBS is crucial for translation initiation and gene expression. The rational design of RBS faces challenges due to the increasing demand for larger libraries, cumbersome experimental procedures, and complex thermodynamic analyses [25,70,71]. To address these challenges, Zhang et al. proposed a machine learning-guided DBTL cycle method for designing bacterial RBS, using the Bandit algorithm to design RBS and the Gaussian process regression (GPR, a nonparametric Bayesian regression method that provides probabilistic predictions of the output by assuming a Gaussian process prior over functions, allows it to capture uncertainty and make predictions with confidence intervals) algorithm to predict the translation initiation rate (TIR) of the designed RBS [52]. The method showed that 34% of the designed RBSs had TIR values not lower than the standard RBS, demonstrating AI algorithms’ potential in RBS sequence design [52]. Subsequently, Simon et al. used the DNA phenotyping method uASPIre (ultra-deep Sequence-Phenotype Interrelationship acquisition) and Next Generation Sequencing (NGS) technology to obtain large datasets of sequence-activity associations [53]. They then trained a CNN model based on these datasets to achieve a precise prediction of RBS activity with an accuracy of 0.927 [53]. This provided an efficient new approach for the precise prediction of RBS activity.

### 2.4. AI-Assisted Design of Protein Sequences and Structures and Prediction of Functional Activity

The de novo design of proteins based on deep learning algorithms can generate novel proteins with expected functions and has been widely applied in protein engineering and synthetic biology [72,73,74,75]. AI-based protein design can be categorized into structure-based design [76,77] and direct sequence design [57,78,79,80]. Despite the rapid accumulation of protein sequence and structure data, the limited number of protein structure types and the vast sequence space remain significant challenges. Using limited data to understand protein folding principles and optimize sequences is a key bottleneck. To overcome these challenges, Karimi et al. used a WGAN (an improved version of GAN by using the Wasserstein distance as a loss function, which enhances training stability and generates higher-quality data) model to obtain low-dimensional representations of the protein folding space [55]. Then, they predicted the structures of the generated sequences based on the Rosetta predictor, achieving high TM scores (TM > 0.5), and indicating accurate folding of AI-designed proteins [55]. Recent studies also focus on using AI to explore sequence space and design proteins directly [72,78]. ProteinGAN, developed by Donata et al., explored the complex multidimensional amino acid sequence space and learned the diversity of natural sequences, generating new sequences with natural physical characteristics [54]. Experimental validation showed high matrix similarity (88%) between the generated and natural sequences, with 24% functionality, indicating that ProteinGAN successfully captured the local amino acid relationships in protein sequences [54]. This demonstrates the potential of AI algorithms to rapidly design diverse functional proteins within a limited sequence space.

**Figure 5:**
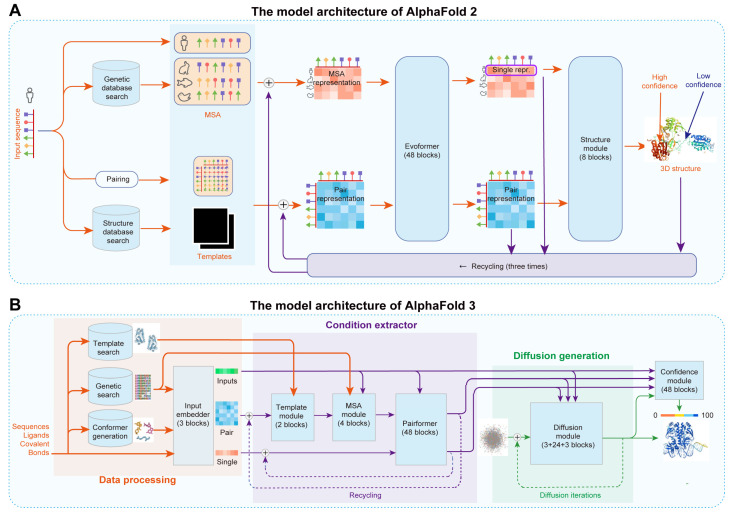
The model architecture of AlphaFold 2 and 3: (**A**) AlphaFold 2 achieves end-to-end prediction of protein 3D structures from protein sequences. The model’s framework is divided into three main steps. First, the protein sequence is input into the model and searched against gene and structure databases to obtain homologous sequences and template structures through MSA. Next, the predicted sequence, MSA sequences, and template structures are embedded and processed by the Evoformer module to generate MSA representation and pair representation. Finally, the first row of the MSA representation and the pair representation are used as inputs for the structure module to predict the 3D structure of the protein sequence. repr., representation. (**B**) Accurate structure prediction of biomolecular interactions based on AlphaFold 3. The AlphaFold 3 model consists of three main steps: data processing, condition extraction, and diffusion generation. First, the required structural prediction information is inputted. Based on this input, the system searches and generates data from databases, deriving information from genetic searches, template searches, and conformer generation. The input information and derived reference information are then fed into the Input Embedder, which performs initial encoding to obtain inputs, single representations, and pair representations. Then, a condition extractor with a recycling mechanism is used to integrate template and MSA information into the pair representation through the Template and MSA Module. The Pairformer then merges and refines the single representation and pair representation information. The inputs, single representations, and pair representations are used as conditions for the diffusion model, which constrains and controls the denoising process to generate refined results. Finally, the predicted results are fed into the Confidence Head to predict the confidence level of the structural prediction.

AlphaFold has revolutionized protein structure prediction based on amino acid sequences [56,78,79,81]. For example, AlphaFold 2, developed by Jumper et al., combines multiple sequence alignments (MSA) and neural networks to achieve near-experimental accuracy in predicting 3D protein structures (Figure 5A) [56]. The model integrates evolutionary, physical, and geometric information, resulting in significantly improved median backbone accuracy as demonstrated in the 14th Critical Assessment of Protein Structure Prediction (CASP14) [82]. AlphaFold 2 achieved a high TM-score of over 0.78 [56]. Despite its advancements, AlphaFold 2 faced challenges such as limited accuracy in predicting complex biomolecular interactions and computational efficiency. To address these challenges, Abramson et al. developed AlphaFold 3, which integrates a diffusion-based architecture to predict the joint structure of complexes, including proteins, nucleic acids, and small molecules, improving accuracy by reducing the complexity of MSA processing and directly predicting raw atom coordinates through a diffusion module (Figure 5B) [57]. AlphaFold 3 achieved an unprecedented accuracy of over 0.8 across various benchmarks, such as protein–ligand and protein–nucleic acid interactions [57]. Thus, AI algorithms provide a crucial direction for the de novo design of proteins.

**Figure 6:**
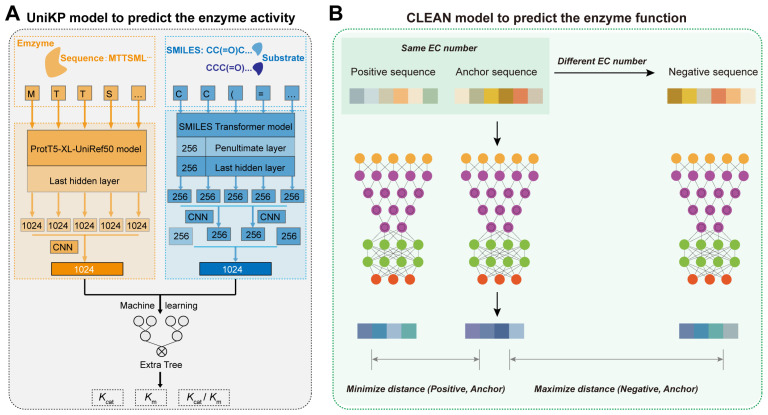
AI-derived prediction of enzyme activity and function: (**A**) The overview of UniKP to predict the enzyme kinetic parameters. First, the pretrained language model, ProtT5-XL-UniRef50, is used to encode enzyme information. Each amino acid is transformed into a 1024-dimensional vector in the last hidden layer. These vectors are then averaged using mean pooling to generate a 1024-dimensional vector representing the enzyme. Next, the pretrained language model, the SMILES transformer, is used to encode substrate information. The substrate structure is converted into a Simplified Molecular Input Line Entry System (SMILES) representation and fed into the SMILES transformer to generate a 1024-dimensional vector. This vector is created by concatenating the average and max pooling of the last layer and the first outputs of the last and penultimate layers. Finally, an interpretable Extra Trees model based on machine learning uses the concatenated representation vectors of the enzyme and substrate as input to predict the *k*_cat_, *k*_m_, or *k*_cat_/*k*_m_ values. (**B**) The overview of CLEAN for the prediction of enzyme function. During training, positive and negative samples are selected based on EC numbers. The input sequences are embedded and processed through a neural network. The warm-colored grid series represents the embeddings of the input sequences from ESM-1b. Similarly, the embeddings obtained from the supervised contrastive learning neural network are depicted in cool colors.

AI algorithms also enhance enzyme activity and function prediction. For enzyme activity prediction, Li et al. developed DLKcat based on CNN and Graph Neural Network (GNN, a deep learning model designed to process and analyze data structured as graphs, consisting of nodes and edges), achieving 0.71 accuracy in predicting the catalytic constant *k*_cat_ [58]. Yu et al. developed the UniKP framework based on pretrained language models, improving the prediction accuracy of *k*_cat_ to 0.85 and achieving 0.73 and 0.81 accuracy in predicting the *k*_m_ and *k*_cat_/*k*_m_, respectively (Figure 6A) [59]. For enzyme function prediction, previous methods often relied on sequence similarity and homology, and some model-based prediction methods were limited by small and imbalanced datasets. To address this problem, Yu et al. developed the CLEAN model using a contrastive learning framework to predict the catalytic functions of different enzymes (Figure 6B) [60]. This model treats the four-digit code of known enzymes as a matrix and uses Euclidean distance to represent functional similarity between different enzymes, ultimately outputting a ranked list of enzyme functions by probability [60]. This approach achieved a precise prediction accuracy of enzyme functions, with a prediction accuracy of over 0.86 [60]. Therefore, AI models provide a new direction for high-precision prediction of enzyme activity and function.

## 3. Optimizing the TFB Response Performance Based on AI-Designed Biological Elements

The optimization of TFB response performance based on the rational design of bio-elements is a key direction in current research. Bio-elements such as promoters, RBS, and transcription factors are crucial regulatory targets in the study of TFB response performance [35,83,84]. AI algorithms have emerged as a novel method for the rational design of bio-elements to achieve optimal TFB response performance.

### 3.1. AI-Designed Promoters for Regulating TFB Response Performance

Promoters can regulate TFB response performance by controlling the transcription rate of transcription factors and reporter proteins [36,85]. Promoter engineering has been widely used to regulate the dynamic range and sensitivity of TFBs [44,86,87]. Strategies for fine-tuning TFB response performance include modifying specific sites of transcription factor-responsive promoters and performing site-directed mutation on promoters [44,86,88]. However, traditional promoter engineering to optimize TFB response performance is often time-consuming, labor-intensive, costly, and limited TFB application scope. In addition, the numerous combinations of promoter motifs can affect TFB response performance due to various factors such as the bio-element activity, the metabolism, and the growth of host cells. Thus, traditional trial-and-error methods face significant challenges in regulating TFB response performance.

**Figure 7:**
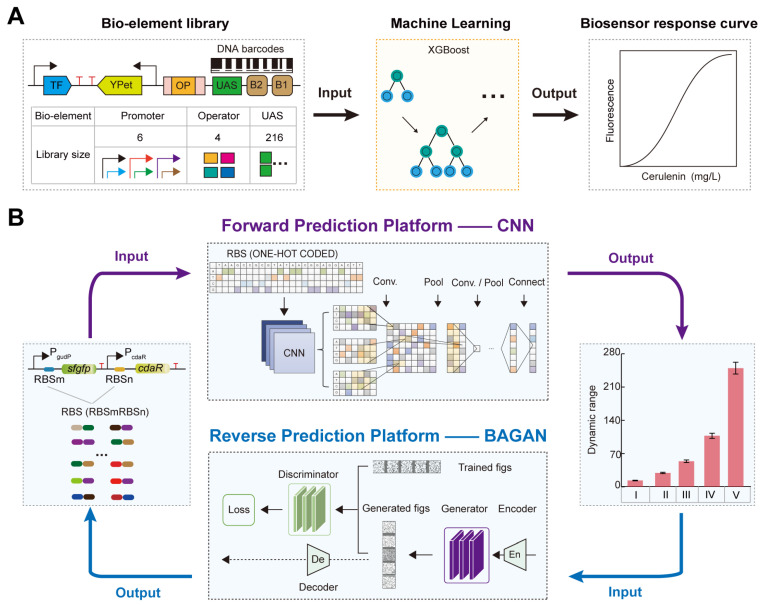
AI-designed bio-elements to fine-tune the TFB response performance: (**A**) The regulation of the cerulenin biosensor response curve based on the XGBoost model. (**B**) The forward–reverse prediction platform to fine-tune the TFB dynamic range. The forward engineering platform to precisely predict the TFB dynamic range based on the CNN model. The reverse engineering platform to rationally design the RBS with the desired TFB dynamic range based on the BAGAN-GP model.

To address these challenges, Zhou et al. synthesized a large dataset of gradient intensity promoters based on DNA barcode technology to construct a TFB library (Figure 7A) [89]. They characterized the TFB response curves using fluorescence-activated cell sorting (FACS) and NGS sequencing (FACS-seq) technologies [89]. Subsequently, they used the XGBoost machine learning model to achieve accurate genotype-to-phenotype predictions [89]. Finally, they experimentally validated the sequences with superior performance based on the prediction results, obtaining a malonyl-CoA biosensor with a maximum dynamic range of 6.38 [89]. This provides an efficient and cost-effective new approach for regulating and optimizing TFB response performance.

### 3.2. AI-Designed RBS for Regulating TFB Response Performance

RBS can regulate the TFB dynamic range by modulating the translation levels of transcription factors and reporter genes as well as protein folding [36,46]. Although RBS engineering has been widely applied in research on regulating the TFB dynamic range [44,90,91], obtaining the corresponding TFB dynamic range for different RBSs still relies on time-consuming and costly experimental studies. Moreover, there is a lack of precise prediction techniques to explore the relationship between RBS sequences and TFB dynamic range. To solve these problems, Ding et al. developed a platform using a deep learning model, CNN, for intelligent prediction of TFB dynamic range based on RBS sequences (Figure 7B) [46]. They first obtained large datasets of glucarate biosensor dynamic range and RBS associations using DNA microarray and FACS-seq technology [46,92]. Then, they built the CLM-RDR platform based on a CNN model to accurately predict the glucarate biosensor dynamic range from RBS sequences, achieving a prediction accuracy of 0.86 [46]. The CLM-RDR platform simplified the workload of the DBTL cycle and enabled precise regulation of TFB dynamic range based on AI-screened RBS sequences.

Furthermore, to rationally design RBS with a desired TFB dynamic range, Ding et al. developed a forward and reverse engineering platform for TFB intelligent design based on CNN and GAN-derived models (Figure 7B) [93]. The forward engineering used the Wasserstein GAN model with gradient penalty (WGAN-GP, an improved version of the Wasserstein GAN that incorporates a gradient penalty term to enforce the Lipschitz constraint, leading to more stable training and better quality generated data compared with the original WGAN) to generate a large functional RBS dataset and predicted the TFB dynamic range from the generated RBS using a CNN model, achieving a prediction accuracy of 0.98 [93]. The reverse engineering used the balanced GAN model (BAGAN-GP, a GAN model designed to restore data balance from unbalanced data sets, incorporating a gradient penalty to improve training stability and the quality of generated data) to de novo design RBS sequences based on a given TFB dynamic range, with a design accuracy of 0.82 [93]. These results indicate that deep learning algorithms have become a crucial tool for the rational design of RBS to optimize TFB dynamic range by exploring the relationship between genotype and phenotype.

### 3.3. AI-Optimized Transcription Factor Regulating the Dynamic Range of TFB

Transcription factors (TFs) are protein molecules that regulate the expression of target genes. Research has shown that the expression levels of TFs and their binding affinity to ligands or target sequences are critical factors affecting TFB response performance. Low TF expression levels can reduce TFB sensitivity and dynamic range, while excessively high TF expression levels can permanently activate or inhibit target gene expression [94]. Additionally, the binding ability of TFs to ligands or DNA affects TFB fluorescence output and dynamic range [85]. Moreover, the regulatory patterns of TFs and metabolites within cells are key factors affecting TFB response performance [35]. Trabelsi et al. constructed a TFB library that included gradient concentrations of the FdeR, the number of binding sites for the activation complex, and plasmid copy numbers [95]. Using a Hill function fitting model, they analyzed the impact of FdeR expression levels and FdeR-ligand binding affinity on TFB response performance, successfully increasing the dynamic range of a naringenin TFB to 60 [95]. This provided new insights for TF design and TFB response performance regulation.

Currently, research on rationally designed TF to regulate TFB response performance has not fully integrated AI algorithms. However, deep learning models have shown excellent performance in analyzing TF characteristics and TFBS interactions [96,97,98]. Simultaneously, AI algorithms like AlphaFold for designing functional proteins have created new opportunities for constructing desired TF. Thus, using AI algorithms to optimize and design TFs provides new possibilities for regulating TFB response performance.

## 4. Applications of Optimized TFB

**Figure 8:**
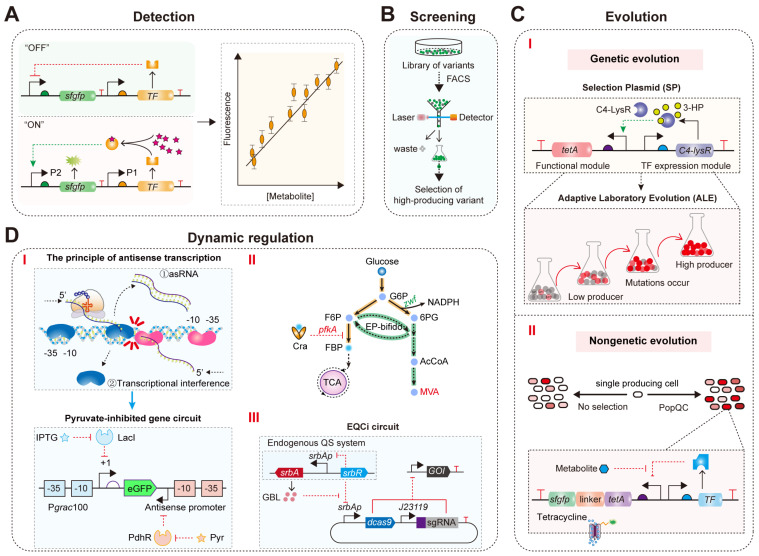
The applications of TFB in metabolic engineering and synthetic biology: (**A**) TFB–derived metabolite concentration detection in real time. The pink stars represent metabolites. (**B**) TFB–derived high-throughput screening of high-titer strain. (**C**) TFB–derived direction evolution of high producer. (**I**) Schematic diagram of using a selection plasmid (SP) as a synthetic biosensor to achieve Adaptive Laboratory Evolution (ALE). The plasmid includes an expression module for the transcription factor C4–LysR and a functional module containing the selection marker *tetA*; (**II**) The design principle of PopQC. PopQC endows nongenetic high–performance cells with a growth advantage, increasing their proportion within the overall population. A metabolite–responsive transcription factor regulates the expression of the tetracycline efflux protein (encoded by tetA). In the presence of tetracycline, high–performance cells outcompete low–performance cells and dominate the population. (**D**) TFB–derived dynamic regulation of microbial intracellular metabolism. (**I**) The schematic diagram of antisense transcription and construction of pyruvate–inhibited gene circuit. The blue and pink irregular patterns represent RNA polymerase initiating transcription from the sense and antisense promoter, respectively. The constitutive promoter (red) at the 3′ end of eGFP suppresses gene expression by triggering RNA polymerase at the sense promoter Pgrac100 (blue). Pgrac100, an IPTG–inducible promoter; LacI, a transcriptional regulator in the *E. coli* lactose metabolism pathway; blue box, the core region of the sense promoter; red box, the core region of the antisense promoter; PdhR, a pyruvate–responsive transcriptional regulator. (**II**) Dynamic control of *pfkA* in EP–bifido strain using a glycolytic flux biosensor for high MVA production. (**III**) The architecture of the genetic circuit based on EQCi. The EQCi genetic circuit is constructed using pSET as the backbone, with the expression of dCas9 driven by the *srbAp* promoter and the transcription of sgRNA targeting the gene of interest (GOI) driven by the strong synthetic promoter J23119 from *E. coli*.

In recent years, TFBs have gained significant attention in the production of compounds within microbial cell factories [35]. The primary applications of TFBs in metabolic engineering include (Figure 8): (1) detection of metabolite concentrations [99]; (2) high-throughput screening of high-yield strains for target metabolites [37,43,100]; (3) directed evolution [37,100,101]; and (4) dynamic regulation of microbial intracellular metabolism [37,102,103,104]. Optimized TFBs with superior response performance are crucial for enhancing the robustness and reliability of these applications.

### 4.1. Real-Time Detection of Target Metabolite Concentrations

Real-time detection of intracellular metabolite concentrations is crucial for optimizing cellular biosynthetic processes. Recently, TFBs have been utilized for this purpose (Figure 8A). For example, Baumann et al. developed a TFB in *Saccharomyces cerevisiae* (*S. cerevisiae*) based on the transcription factor War1p, the PDR12 promoter responsive to short-chain and medium-chain fatty acid (SMCFA), and the reporter gene *gfp* [99]. This system allows for easy and rapid detection of SMCFA, serving as an alternative to traditional gas chromatography methods [99]. The TFB exhibited linear responses to hexanoic, heptanoic, and octanoic acids within the concentration ranges of 0.01–2.00 mM (R^2^ = 0.98), 0.01–1.50 mM (R^2^ = 0.99), and 0.01–0.75 mM (R^2^ = 0.99), respectively [99]. Consequently, this TFB has the potential to significantly accelerate the engineering of cell factories for the production of various SMCFAs. However, current detection systems often have limited detection ranges and fail to show concentration-dependent fluorescence changes when target metabolite concentrations exceed millimolar levels, thus restricting their further application in metabolic engineering and synthetic biology [105,106]. Therefore, designing TFBs with the desired performance is crucial for detecting higher metabolite concentrations.

### 4.2. High-Throughput Screening of High-Titer Strains for Target Metabolites

TFBs are not only used to detect intracellular metabolite concentrations but are also widely applied in the screening of high-titer strains for target metabolites [36,100,107]. TFBs can be used in conjunction with FACS to rapidly screen high-titer strains from extensive libraries by detecting the output signal of fluorescent reporter genes (Figure 8B) [37,83,100]. For example, Kortmann et al. constructed a TFB responsive to L-lysine based on LysG and used it with a FACS screening system to identify pyruvate carboxylase mutants in *C. glutamicum* [108]. This approach improved the ability of *C. glutamicum* to produce L-lysine from glucose [108]. When *C. glutamicum* produces high levels of L-lysine, LysG senses the L-lysine concentration and activates the expression of a fluorescent protein, generating a fluorescence signal [108]. By screening the pyruvate carboxylase mutant library, two mutants that significantly increased L-lysine production in host cells were identified, leading to L-lysine levels increasing by 6% and 14%, respectively [108]. Similarly, Ding et al. developed a TFB responsive to glucaric acid (GA) based on CdaR and used it with FACS to screen for *myo*-inositol oxygenase (MIOX) mutants with high stability and activity, a key rate-limiting enzyme in the GA biosynthesis pathway [92]. This approach increased GA titer to 5.52 g/L in 5 L fermenter cultures, the highest titer reported in *E. coli* to date [92]. These successful cases demonstrate that TFBs can be effectively integrated with mainstream high-throughput screening methods. However, the expression of fluorescent proteins often imposes a metabolic burden on cells, affecting cell growth and potentially leading to bias in FACS screening [109]. Therefore, using antibiotic-resistant genes instead of fluorescent proteins for screening high-titer strains may alleviate this issue.

### 4.3. Directed Evolution

TFB-mediated directed evolution is a powerful strategy for the efficient production of target metabolites [40,110,111]. Optimized TFBs can enrich high-titer strains by responding to target metabolites and activating or inhibiting downstream gene expression [101,112,113,114]. For example, Seok et al. developed a synthetic biosensor responsive to 3-hydroxypropionic acid (3-HP) based on the C4-LysR biosensor and the TetA bioselector (Figure 8C(I)) [101]. Using a glycerol-dependent 3-HP production pathway as a model system, they performed adaptive laboratory evolution (ALE) to identify the optimal flux redistribution between the 3-HP biosynthesis pathway and the central carbon metabolism pathway, increasing the 3-HP titer and reducing acetate accumulation by alleviating overflow metabolism [101]. These results demonstrate that whole-genome evolution using synthetic biosensors can lead to effective carbon flux rewiring. Additionally, Shen et al. used a 4-hydroxyphenylacetic acid (4HPAA) biosensor combined with atmospheric and room temperature plasma (ARTP) mutagenesis and ALE to successfully obtain strains with high 4HPAA titer and tolerance [115]. The strains maintained genetic stability after 25 generations of genome shuffling [115]. Ultimately, strain GS-2-4 produced 25.42 g/L 4HPAA in a 2 L fed-batch culture bioreactor [115]. These results indicate that the strain has long-term genetic stability and high production levels, making it a potential candidate for industrial applications. Moreover, Tong et al. constructed a TFB responsive to (2S)-naringenin in *E. coli* based on TtgR [116]. Through directed evolution and saturation mutagenesis, they identified a chalcone synthase (CHS) mutant, *Sj*CHS1^S208N^, with 2.34-fold increased catalytic activity [116]. Fermentation in a 5 L bioreactor increased the de novo (2S)-naringenin concentration to 2513 ± 105 mg/L, the highest concentration reported in a stirred batch bioreactor to date [116]. Overall, these directed evolution strategies can be broadly applied to engineer biochemical production pathways without the need for labor-intensive procedures.

In addition, natural heterogeneity caused by nongenetic factors exists between cells at the protein and metabolite concentration levels [117]. Previous studies have shown that genetic heterogeneity in industrial fermentation processes can lead to production burdens due to metabolic load and toxicity, which negatively impact titers [117]. To mitigate the effects of fermentation heterogeneity on metabolite production, Xiao et al. developed a Population Quality Control (PopQC) system based on the FadR biosensor and TetA bioselector (Figure 8C(II)) [118]. This system continuously enriches high-producing cells and eliminates inefficient ones, resulting in a threefold increase in fatty acid production titer [118]. Similarly, Ding et al. constructed a PopQC system for GA production based on the CdaR biosensor and TetA [92]. High intracellular GA levels trigger the GA biosensor to express TetA, providing a growth advantage to high GA-producing cells under tetracycline selective pressure, ultimately increasing the GA production titer to 5.52 g/L in a 5 L fermenter [92]. Therefore, TFBs are invaluable for studying and controlling metabolic heterogeneity.

### 4.4. Dynamic Regulation of Microbial Intracellular Metabolism

Using TFBs to dynamically regulate intracellular gene metabolism levels in response to intracellular metabolic states can simulate the naturally occurring metabolic regulatory networks of microbes. This approach can prevent the excessive accumulation of toxic metabolic intermediates and balance the supply of precursors needed for cell growth with the biosynthesis of target metabolites [103,104,119,120]. For example, Zhou et al. designed a TFB based on FdeR and PadR that responds simultaneously to (2S)-naringenin and p-coumaric acid [121]. They used this biosensor to control the synthesis and consumption of malonyl-CoA [121]. Low concentrations of (2S)-naringenin direct malonyl-CoA towards the fatty acid biosynthesis pathway, promoting cell growth [121]. High concentrations of (2S)-naringenin inhibit the fatty acid biosynthesis pathway, slowing cell growth and increasing the availability of malonyl-CoA for producing more (2S)-naringenin [121]. Ultimately, this multilayer dynamic regulatory network increased the titer of naringenin by 8.7-fold [121]. This indicates that dynamic regulation is a promising strategy for fine-tuning metabolic flux in microbial cell factories. However, these synthetic regulatory systems are rarely developed for central carbon metabolites and can only activate or inhibit the expression of target genes, failing to achieve dual-functional dynamic regulation of metabolic pathways. To enable dynamic dual control (activation and inhibition) for central metabolism, Xu et al. constructed a bifunctional pyruvate-responsive biosensor using the PdhR from *E. coli* and an antisense transcription-based “NOT” gate for signal conversion (Figure 8D(I)) [102]. By dynamically upregulating the *ino1* gene and downregulating the *zwf* and *pgi* genes, they increased glucaric acid production from 207 mg/L to 527 mg/L [102]. Additionally, Zhu et al. designed and constructed a bifunctional glycolytic flux biosensor (Figure 8D(II)) [122]. They modified promoters and transcriptional regulators to obtain highly responsive activation and inhibition biosensors for dynamic control of glycolytic flux [122]. Using this biosensor, they upregulated the expression of *zwf*, encoding glucose-6-phosphate dehydrogenase, and downregulated *pfkA*, encoding phosphofructokinase (Figure 8D(II)) [122]. This ultimately increased the mevalonate production titer in *E. coli* to 111.3 g/L in a 1 L fermenter [122]. Thus, bifunctional biosensors are effective tools for dynamically controlling central metabolism in microbial cell factories.

However, the currently available dynamic regulatory elements are very limited, and many metabolites lack specific responsive transcription factors. Therefore, developing convenient, universal, and self-driven dynamic control systems is of great significance for the efficient biosynthesis of target metabolites in microbes. For example, Tian et al. developed a novel dynamic regulation system, EQCi (Endogenous Quorum-sensing (QS) system with Clustered Regularly Interspaced Short Palindromic Repeats interference (CRISPRi)), in *Streptomyces* (Figure 8D(III)) [123]. This system uses a γ-butyrolactone (GBL) signal molecule-responsive promoter to drive the expression of the dCas9, coupling the QS system with gene transcription inhibition technology (CRISPRi) [123]. It allows for fully automated and precise dynamic control of multiple genes in the metabolic pathway [123]. Using the EQCi system, they constructed a rapamycin-producing recombinant strain [123]. By downregulating key genes in the tricarboxylic acid cycle, fatty acid biosynthesis, and shikimate pathways, they increased the precursor supply for rapamycin biosynthesis, improving the production titer [123]. They then used the EQCi system for combined intervention in the metabolic flux of the three pathways and fine-tuning of control strength at each node, resulting in an optimized engineered strain with a rapamycin titer of 1836 ± 191 mg/L, approximately 6.6 times higher than the natural strain [123]. This indicates that the EQCi system effectively balanced the metabolic flux distribution between primary metabolism and product biosynthesis (secondary metabolism), providing an efficient and universal optimization strategy for constructing cell factories of important secondary metabolites derived from *Streptomyces*.

## 5. Conclusions and Perspective

To deepen our understanding of how bio-elements regulate cellular metabolism and apply these insights to metabolic engineering and synthetic biology, researchers have increasingly focused on the rational design and activity prediction of bio-elements, as well as the optimization of TFB response performance using rationally designed bio-elements. The integration of AI technology with bio-element engineering has matured, leading to the widespread application of machine learning and deep learning algorithms in bio-element research. AI algorithms have shown excellent performance in the high-quality design and precise prediction of nucleic acid and protein elements, as well as the efficient optimization of TFB response performance [124,125]. Deep generative models, such as GANs, have emerged as important tools in designing expected nucleic acid and protein sequences due to their ability to de novo generate novel sequences [126]. The remarkable application of AI in the field of bio-elements demonstrates its powerful potential in uncovering biological characteristics and designing biological systems. This paves the way for scalable, automated, engineered, and end-to-end intelligent prediction and design.

AI model training often depends on substantial high-quality, standardized biological data, especially for deep learning models [127]. For example, extensive datasets that associate bio-element sequences with their activity or structure are indispensable in predicting the activity or structure of bio-elements. However, obtaining large amounts of novel high-quality biological data is often costly, and the data frequently contain indistinct features and significant noise, which complicates model performance optimization. Therefore, the lack of high-quality biological data is a significant challenge that must be addressed for the effective application of AI in bio-element research. Future deep learning models may need to extract features deeply and achieve precise predictions and high-quality bio-element generation from relatively small datasets. Additionally, AI algorithms face diverse challenges in model construction and optimization across biological problems and researchers. On one hand, the functionalities and characteristics of the selected models need to match the specific data structures and research objectives. For example, RNN and Transformer models can handle sequence data [128,129], while GCN models excel at processing topological graph structures [130,131]. CNN models are adept at handling prediction tasks, and GAN models are proficient in de novo design of bio-elements. On the other hand, high-performing AI models often rely on the selection of the optimal evaluation metrics and fine-tuning of model parameters. Thus, the deep integration of AI algorithms with bio-element research requires researchers to have a solid understanding of interdisciplinary knowledge.

Due to the high complexity of cellular systems, data feature extraction techniques must be capable of precisely capturing highly ambiguous and low-precision features. AI models must not only achieve high performance in actual prediction and design tasks but also possess sufficient generalization ability and interpretability to handle diverse and challenging tasks. However, many models with strong predictive or generative capabilities still have problems with the biological interpretability of their computational processes and outputs. Therefore, future applications require the integration of AI model mechanisms and biological theories to enhance model performance while improving interpretability. In terms of model interpretability, Zheng et al. developed the NeuronMotif neural network interpretation algorithm, which can learn and summarize gene regulatory sequence coding rules from neurons, providing a method to interpret the pattern recognition of CNN models [132]. As interdisciplinary fields continue to develop, an increasing number of standardized databases are becoming available on shared platforms. The future emergence of the World Wide Web will further reduce the difficulty of acquiring high-quality data. The advent of deep learning models such as EfficientNet [133], Swin-Transformer [134], and LLMs [135] will further enhance the performance and efficiency of AI algorithms in the design and prediction of bio-elements. 

In the future, several key areas will shape the field of AI-assisted rational design and activity prediction of bio-elements for optimizing TFB. Emerging AI technologies, such as deep reinforcement learning [136,137,138], unsupervised learning techniques [139], and advanced neural network architectures [140], will provide powerful tools for bio-element design. Integrating AI with other scientific disciplines, such as systems biology and bioinformatics, will enhance our understanding of complex biological systems and improve the precision and effectiveness of bio-element design. Potential applications in medicine [141,142,143,144,145,146], agriculture [147], and environmental science [148,149] will expand as AI-designed enzymes and metabolic pathways revolutionize drug discovery [150] and biomanufacturing processes. Addressing challenges in data acquisition, model interpretability, and ethical considerations will be crucial. Future research should focus on developing standardized data-sharing protocols, enhancing model transparency, and establishing ethical guidelines for AI applications in biology. Generating high-quality data and refining AI models to handle biological complexity will be essential for advancing AI-assisted bio-element design. This includes optimizing TFB, enhancing the robustness and reliability of TFB applications, and ensuring that AI-driven solutions meet the diverse needs of synthetic biology. By tackling these challenges and leveraging the full potential of AI, researchers can significantly advance the field, making AI an indispensable tool in bio-element research and applications.

In conclusion, AI algorithms have already made significant contributions to the field of bio-elements. Although challenges remain in data acquisition, model construction, and optimization, execution of diverse tasks, and model interpretability, machine learning and deep learning methods based on AI algorithms remain indispensable tools. These tools are crucial not only for the rational design and activity prediction of bio-elements but also for optimizing TFB response performance and enhancing the robustness and reliability of TFB applications. By consolidating current research, highlighting innovative AI-driven solutions, and addressing existing challenges, this review demonstrates how AI can transform synthetic biology by improving precision, efficiency, and practicality in bio-element design. These insights offer significant advancements for both academic research and practical applications in biotechnology. Future developments in AI models will continue to drive progress in synthetic biology, ensuring more robust and reliable outcomes.

## Figures and Tables

**Table 1:** Challenges and strategies in traditional and AI-based bio-element design and activity prediction.

Bio-Element	Function	Challenge	Strategy		Accuracy		References
			Traditional	AI	Traditional	AI	
Promoter	Rational design and activity prediction	Small library, vast sequence space	Experimental method	GAN, CNN	ns	0.7	[31]
				DeepSEED (GAN, LSTM)		0.78	[47]
	Activity prediction	High prediction cost and low accuracy	CHIP-seq, RNA-seq	XGBoost		0.88	[48]
			CHIP-seq, RNA-seq	iPro-GAN		0.92	[49]
Enhancer	Rational design and activity prediction	Unclear motif syntax relationships, inadequate compatibility between motifs, and limited applicability	Experimental method	DeepSTARR (CNN), GAN	ns	0.74	[50]
	Activity prediction		CHIP-seq, RNA-seq	iEnhancer-DCLA (CNN, BiLSTM, Attention)		0.83	[51]
RBS	Activity prediction	Demand for larger libraries, cumbersome experimental procedures, and complex thermodynamic analysis data	Experimental method	GPR, Bandit	ns	34% high TIR	[52]
			Ribosome loading, DNA methylation, NGS	CNN		0.927	[53]
Protein	Rational design	Limited sequence space	ns	ProteinGAN (GAN)	ns	0.88	[54]
	Rational design and activity prediction	Limited protein structure types and vast sequence space	Experimental method	WGAN, Rosetta		TM > 0.5	[55]
	Activity prediction	Low accuracy		AlphaFold 2		TM > 0.78	[56]
		and limited accuracy for complex interactions		AlphaFold 3		>0.8	[57]
	Enzyme catalytic constant prediction	Low accuracy		DLKcat (CNN, GNN)		0.71 (*k*_cat_)	[58]
				UniKP (pretrained language models)		0.85 (*k*_cat_), 0.73 (*k*_m_), 0.81 (*k*_cat_/*k*_m_)	[59]
	Enzyme function prediction	Small and imbalanced datasets		CLEAN (contrastive learning framework)		0.86	[60]

ns, not specified.

## Data Availability

Date sharing not applicable.

## Abbreviations

AI: Artificial intelligence; Bio-elements: Biological elements; RBS: Ribosome binding site; TF: Transcription factor; CNN: Convolutional neural network; RNN: Recurrent neural network; GAN: Generative adversarial network; DBTL: Design–Build–Test–Learn; TFB: Transcription-factor-based biosensor; LSTM: Long short-term memory; XGBoost: extreme gradient boosting; TFBS: Transcription factor binding site; PCC: Pearson correlation coefficient; TIR: Translation initiation rate; GPR: Gaussian process regression; NGS: Next generation sequencing; GNN: Graph neural network; WGAN: Wasserstein GAN; FACS: Fluorescence-activated cell sorting; WGAN-GP: WGAN with gradient penalty; BAGAN: Balanced GAN; SMCFA: Short-chain and medium-chain fatty acid; GA: Glucaric acid; MIOX: *Myo*-inositol oxygenase; PopQC: Population quality control.
